# Patient-Reported Symptom Relief Following Medical Cannabis Consumption

**DOI:** 10.3389/fphar.2018.00916

**Published:** 2018-08-28

**Authors:** Sarah S. Stith, Jacob M. Vigil, Franco Brockelman, Keenan Keeling, Branden Hall

**Affiliations:** ^1^Department of Economics, The University of New Mexico, Albuquerque, NM, United States; ^2^Department of Psychology, The University of New Mexico, Albuquerque, NM, United States; ^3^The MoreBetter Ltd., Washington, DC, United States

**Keywords:** pain, anxiety, depression, cannabis, marijuana, quality of life, symptom management, side effects

## Abstract

**Background:** The Releaf App^TM^ mobile software application (app) data was used to measure self-reported effectiveness and side effects of medical cannabis used under naturalistic conditions.

**Methods:** Between 5/03/2016 and 12/16/2017, 2,830 Releaf App^TM^ users completed 13,638 individual sessions self-administering medical cannabis and indicated their primary health symptom severity rating on an 11-point (0–10) visual analog scale in real-time prior to and following cannabis consumption, along with experienced side effects.

**Results:** Releaf App^TM^ responders used cannabis to treat myriad health symptoms, the most frequent relating to pain, anxiety, and depressive conditions. Significant symptom severity reductions were reported for all the symptom categories, with mean reductions between 2.8 and 4.6 points (ds ranged from 1.29–2.39, *p*s < 0.001). On average, higher pre-dosing symptom levels were associated with greater reported symptom relief, and users treating anxiety or depression-related symptoms reported significantly more relief (ps < 0.001) than users with pain symptoms. Of the 42 possible side effects, users were more likely to indicate and showed a stronger correlation between symptom relief and experiences of positive (94% of sessions) or a context-specific side effects (76%), whereas negative side effects (60%) were associated with lessened, yet still significant symptom relief and were more common among patients treating a depressive symptom relative to patients treating anxiety and pain-related conditions.

**Conclusion:** Patient-managed cannabis use is associated with clinically significant improvements in self-reported symptom relief for treating a wide range of health conditions, along with frequent positive and negative side effects.

## Introduction

Medicinal cannabis use is expanding rapidly in the United States, with thousands of new users daily, particularly older patients and people with significant health concerns, treating many different symptoms (Centers for Disease Control and Prevention, 2016; Han et al., 2016). Most patients have a wide variety of medicinal cannabis products available to them, ranging from traditional flower to edibles and tinctures. Naturalistic observational studies are generally well-suited for capturing how patients manage their treatment decisions in real-life, and how patient-managed cannabis therapies may contribute to symptom relief and potential side effects from use. Observational research designs allow patients to use the myriad *Cannabis* strains and cannabis-derived formulations (e.g., concentrates, tinctures, edibles, topicals, suppositories, toothpaste) made at home and/or commercially available and widely used in society, and can incorporate the breadth of health conditions for which medical cannabis has been sanctioned for use at the state-level. Lastly, observational studies also circumvent research barriers associated with cannabis’ Schedule I status under United States federal law, which makes randomized controlled trials (RCTs) challenging to conduct (Stith and Vigil, 2016; National Academies of Sciences, Engineering, and Medicine, 2017).

Since its release in 2016, the commercially developed Releaf App^TM^ application (app; Releaf App, 2018) has been the only publically available, incentive-free patient educational software program designed for recording how individual cannabis usage sessions may correspond to immediate changes in primary symptom intensity levels and experienced side effects. This electronic assessment tool enables patients to monitor and manage their cannabis consumption decisions under naturalistic conditions while avoiding the limitations of retrospective survey collection methods (e.g., memory bias, social desirability effects). We used the Releaf App^TM^ repository of over 2,830 patients and 13,368 individual cannabis administration sessions to examine two research questions: How does cannabis used under naturalistic conditions affect user-experienced symptom relief and side effects? Does the magnitude of experienced symptom relief and the prevalence of side effects vary across symptom categories? The results have clinical relevance for understanding how patient-managed medical cannabis therapies may correspond to changes in symptom intensity and potential side effects among people using cannabis for treating distinct health conditions (Hill and Weiss, 2016; Rubin, 2017).

## Materials and Methods

### Study Design

A naturalistic observational research design, approved by the Institutional Review Board at the University of New Mexico, was used to analyze the Releaf App^TM^ user-submitted data recorded between 5/03/2016 and 12/16/2017. Releaf App^TM^ is a cross-platform (iOS and Android) mobile and tablet app backed by a secure cloud programming interface for capturing, processing, and storing anonymized user data. Out of 4,369 total users and 23,373 user interactions, we included only cannabis consumption sessions with reported starting symptom levels greater than 0 (on a 0–10, 11-point scale) and ending symptom levels reported within 90 min of the start of the session, resulting in a final sample of 2,830 users and 13,638 individual sessions for analysis. The Releaf App^TM^ measures 27 possible negative symptom categories and 42 possible side effects. Symptoms were ultimately derived from qualifying conditions across medical cannabis programs in the United States, along with a few suggested by dispensaries and patients. The side effects (called “feelings” within the app) were crowd-sourced among Releaf App^TM^ developers, beta testers, dispensaries, and patients, and included 19 positive, 12 negative, and 11 context-specific side effects available for selection. **Supplementary Tables S1, S2** in the **Supplemental Appendix** provide descriptive statistics for all symptoms and side effects.

User sessions consist of a series of electronic instructions for recording characteristics of the cannabis medication (e.g., strain, potency, formulation), pre-dosing symptom severity rating along an 11-point visual analog facial pain scale from 0 (no detectable symptom level) to10 (severe), the timing of cannabis consumption, a post-dosing symptom severity rating, and the option to indicate any of the 42 listed side effects at any time during the session. Among our primary sample of users, 2,332 users reported side effects during 10,535 sessions.

### Study Outcomes

Our goal was to calculate changes in patient-perceived symptom severity, the prevalence of positive and negative side effects associated with cannabis consumption, and whether the reported-effects differs depending on the symptom for which users were seeking treatment. We measured changes in symptom relief by subtracting the ending symptom level from the beginning symptom (possible range from -10 to 10). (**Supplementary Figure S1** in the **Supplemental Appendix** provides a frequency table for each level of symptom relief.) Side effects were recorded as {0,1} variables for whether the user selected that side effect from the menu. We categorize the side effects as positive, negative, or context-specific and then convert these categories of side effects into {0,1} outcomes, count outcomes and outcomes measuring the portion of total available side effects in that category a user selected.

### Statistical Analysis

We use means comparisons and least squares regression models to estimate the absolute and relative symptom changes and side effect profiles resulting from the cannabis user sessions. We also created an *adjusted symptom relief profile score*, the mean change in symptom levels plus the absolute number of listed negative side effects, to provide a relative metric of cost-benefit tradeoffs associated with cannabis use. Due to the small user counts for some of the reported symptoms, the large number of possible symptoms, and to facilitate interpretation in our regression analysis, we aggregate the most commonly reported symptoms across three broad symptom categories that included: Anxiety Symptoms (agitation/irritability, anxiety, insomnia, stress, and muscle spasms), Pain Symptoms (ten pain categories), and Depression Symptoms (depression). The remaining types of symptoms are less frequently reported or not clearly categorized. We also report the full regression results for the three categories of side effects (positive, negative, and context-specific) and the sign for regressions of symptom relief on the full range of 42 side effects. Standard errors are clustered at the user level to control for heteroskedasticity and arbitrary correlation.

## Results

**FIGURE 1:**
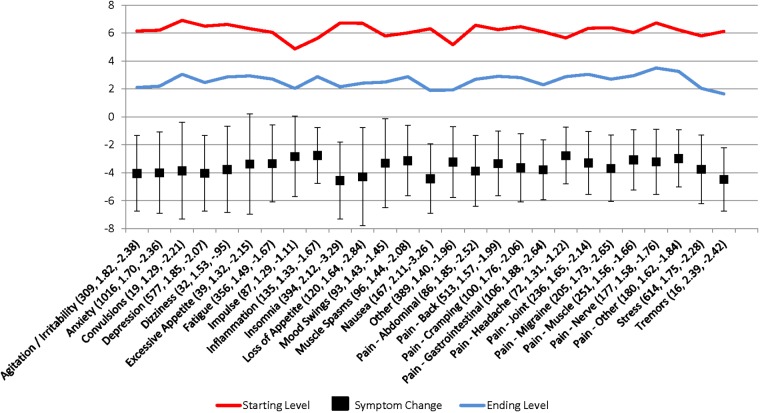
Patient-reported symptom relief following medical cannabis consumption. Values in parantheses are the symptom category sample size, Cohen’s d, and adjusted symptom relief score (symptom relief + number of negative side effects), respectively.

**Figure 1** shows the starting and ending symptom severity levels, the change in levels, the Cohen’s d of the difference, and the adjusted symptom relief profile score for each of the 27 discrete symptom categories. For all symptoms, the null hypothesis that the starting symptom severity level is less than or equal to the ending symptom severity can be rejected at the *p* < 0.001 level. Using the adjusted symptom relief measure (symptom relief plus negative side effects), all but users with convulsions, dizziness, excessive appetite, or tremors experienced a net improvement in their symptom severity levels. Even for these symptoms, the adjusted mean symptom relief score still indicates a net benefit from use and the lack of a statistically significant change likely relates more to the small number of observations rather than the lack of an effect, given that these symptoms together constituted less than 3% of users and less than 1% of our sample. For all other symptoms, the null hypothesis of an increase or no change in the adjusted symptom relief score can be rejected at the *p* < 0.001 level.

**Table 1** provides additional information on starting and ending symptom severity levels, mean symptom relief, and the prevalence of positive, negative, and context-specific side effects by the aggregated symptom categories (anxiety, pain, and depression symptoms). For completeness, we include a fifth column including the remaining discrete symptom categories which did not fall under the three aggregated symptom categories. Little variation exists in starting and ending symptom levels and the symptom relief experienced, with the average user reporting a symptom decrease of 3.7. With regards to side effects, those with depression have a higher probability of reporting negative or context-specific side effects. The most common positive side effects are “relaxed” (64%), “peaceful” (54%), and “comfy” (38%), the most common negative side effects are “dry mouth” (23%), “foggy” (22%), and “forgetful” (13%) and the most common context-specific side effects are “high” (32%), “sleepy” (27%), and “thirsty” (27%).

**Table 1:** Descriptive statistics – symptom levels and experienced side effects.

	Overall	Anxiety symptoms	Pain symptoms	Depression symptoms	Other
N Sessions	13638	5343	4267	1440	2588
N Users	2830	1679	1223	577	1026
Starting symptom level	6.2 ± 2.2	6.2 ± 2.3	6.3 ± 2.0	6.5 ± 2.2	5.8 ± 2.4
Ending symptom level	2.5 ± 2.2	2.2 ± 2.2	3.0 ± 2.1	2.5 ± 2.2	2.4 ± 2.3
Symptom relief	-3.7 ± 2.6	-4.0 ± 2.8	-3.3 ± 2.3	-4.0 ± 2.7	-3.4 ± 2.8
Better	94.2%	94.8%	94.7%	95.4%	91.6%
Same	2.7%	2.4%	2.8%	2.4%	3.2%
Worse	3.1%	2.8%	2.5%	2.2%	5.2%
Any positive side effect	94.4%	94.7%	94.5%	93.9%	94.2%
Any negative side effect	60.0%	60.0%	58.9%	65.5%	58.8%
Any context-specific side effect	76.2%	75.2%	75.9%	80.1%	76.6%
# of positive side effects	4.6 ± 3.2	4.6 ± 3.2	4.4 ± 3.1	4.8 ± 3.4	4.8 ± 3.4
# of negative side effects	1.4 ± 1.7	1.4 ± 1.7	1.3 ± 1.6	1.6 ± 1.9	1.3 ± 1.7
# of context-specific side effects	2.0 ± 1.9	2.0 ± 1.9	1.9 ± 1.9	2.1 ± 1.9	2.0 ± 1.9
% of positive side effects	24%	24%	23%	26%	25%
% of negative side effects	11%	11%	10%	13%	10%
% of context-specific side effects	20%	20%	19%	21%	20%


*Symptoms designated as treatable with benzodiazepines (Anxiety Symptoms) include agitation/irritability, anxiety, insomnia, muscle spasms, and stress. Symptoms associated with Opioid treatment (Pain Symptoms) include all ten pain conditions. Depression is the only symptom designated as treatable with antidepressants.*

**Table 2** examines how symptom relief varies across the broader symptom categories, with the constant representing the mean adjusted symptom change for the omitted category, (patients with pain-related symptoms). The first two regressions shown in **Table 2** indicate that people with anxiety and depression report greater relief from using cannabis than people with chronic pain, and users with higher starting symptom levels report greater symptom relief. (The effects of cannabis on anxiety and depression symptoms are not statistically different from each other, although they are both greater than the effect of cannabis on pain-related symptoms). Negative responses or increases in symptom severity do occur, but the intercept in combination with the starting symptom level predicts that increases in symptom severity levels predominantly occur among users with starting symptoms equal to one. The third column in **Table 2** shows that cannabis is more effective for anxiety and depression symptoms than for pain-related symptoms among patients reporting higher symptom severity levels (A graphical representation of this relationship is presented in **Supplementary Figure S2** in the **Supplemental Appendix**).

**Table 2:** Reported symptom relief for users treating anxiety, pain, and depression.

Outcome = symptom relief			

	(1)	(2)	(3)
Constant (opioid mean)	-3.309^∗∗∗^	1.120^∗∗∗^	0.355^∗∗^
	(-3.459 to -3.160)	(0.804 to 1.436)	(0.034 to 0.675)
Anxiety symptoms	-0.704^∗∗∗^	-0.763^∗∗∗^	0.365^∗^
	(-0.944 to -0.465)	(-0.953 to -0.574)	(-0.062 to 0.792)
Depression symptoms	-0.723^∗∗∗^	-0.563^∗∗∗^	0.643^∗^
	(-1.060 to -0.385)	(-0.817 to -0.310)	(-0.021 to 1.308)
Starting symptom level (1–10)		-0.706^∗∗∗^	-0.582^∗∗∗^
		(-0.757 to -0.656)	(-0.639 to -0.525)
Anxiety^∗^start			-0.181^∗∗∗^
			(-0.259 to -0.102)
Depression^∗^start			-0.189^∗∗∗^
			(-0.305 to -0.074)
Observations	11,050	11,050	11,050
*R*^2^	0.018	0.372	0.377


*Each column represents a separate regression. The omitted category is symptoms treatable with an opioid medication. Robust standard errors are clustered at the user level. The coefficients are reported in line with the variable names with confidence intervals below. Coefficients are reported with 95% Confidence Intervals below. ^∗∗∗^p < 0.01, ^∗∗^p < 0.05, ^∗^p < 0.10.*

In order to take advantage of the full range of symptom categories available to Releaf App^TM^ users, we also ran regressions including dummy variables for each of the symptoms, using back pain as the omitted category. After controlling for starting symptom level, clustering the standard errors at the user level, and using a statistical significance threshold of *p* < 0.05, our results indicate that patients report greater symptom relief for treating agitation/irritability, anxiety, depression, excessive appetite, insomnia, loss of appetite, nausea, gastrointestinal pain, stress, and tremors than they do for treating back pain. Patients reported less symptom relief for treating impulsivity, headache, and nerve pain as compared to relief for treating back pain. The symptom relief for the other discrete symptom categories was indistinguishable from the reported symptom relief associated with back pain.

**Table 3** explores whether patients using cannabis to treat pain, anxiety, or depressive symptoms differ in their experiences of positive, negative, or context-specific side effects. Chows tests (Chow, 1960) showed that users with anxiety-related symptoms are no more or less likely than those with pain symptoms to report any of the three categories of side effects. Individuals with depression, however, are more likely to report negative and context-specific side effects than positive side effects. Higher starting symptom levels are also associated with more negative or context-specific side effect reporting and this relationship persists whether the side effect profile is defined as any of the side effects from that category of side effects, the number of side effects by category, or the percent of possible side effects in a category.

**Table 3:** Differences in side effect profiles across symptom categories.

Outcome = side effect type			

	**Positive**	**Negative**	**Context-specific**
	
	**Any**		
Constant (opioid mean)	0.966^∗∗∗^	0.496^∗∗∗^	0.695^∗∗∗^
	(0.942 to 0.989)	(0.428 to 0.565)	(0.637 to 0.753)
Anxiety symptoms	0.001	0.013	-0.006
	(-0.012 to 0.015)	(-0.033 to 0.059)	(-0.049 to 0.037)
Depression symptoms	-0.006	0.066^∗∗^	0.042^∗^
	(-0.029 to 0.017)	(0.002 to 0.131)	(-0.005 to 0.090)
Starting symptom level	-0.003^∗^	0.015^∗∗∗^	0.010^∗∗^
	(-0.007 to 0.000)	(0.007 to 0.024)	(0.002 to 0.019)

	**Number**		

Constant (opioid mean)	4.583^∗∗∗^	1.081^∗∗∗^	1.652^∗∗∗^
	(4.013 to 5.154)	(0.768 to 1.395)	(1.356 to 1.947)
Anxiety symptoms	0.182	0.077	0.077
	(-0.100 to 0.465)	(-0.104 to 0.257)	(-0.113 to 0.268)
Depression symptoms	0.476^∗^	0.324^∗∗^	0.134
	(-0.010 to 0.962)	(0.053 to 0.596)	(-0.187 to 0.454)
Starting symptom level	-0.035	0.036^∗∗^	0.044^∗∗^
	(-0.142 to 0.072)	(0.000 to 0.072)	(0.003 to 0.085)

	**Percent of possible**		

Constant (opioid mean)	0.241^∗∗∗^	0.083^∗∗∗^	0.165^∗∗∗^
	(0.211 to 0.271)	(0.059 to 0.107)	(0.136 to 0.195)
Anxiety symptoms	0.01	0.006	0.008
	(-0.005 to 0.024)	(-0.008 to 0.020)	(-0.011 to 0.027)
Depression symptoms	0.025^∗^	0.025^∗∗^	0.013
	(-0.001 to 0.051)	(0.004 to 0.046)	(-0.019 to 0.045)
Starting symptom level	-0.002	0.003^∗∗^	0.004^∗∗^
	(-0.007 to 0.004)	(0.000 to 0.006)	(0.000 to 0.009)


*The first panel uses {0,1} outcomes for the presence of side effects in each category, the second uses the count of side effects reported by category, and the third uses the number of reported side effects for each category divided by the total number of possible side effects a user could select in that category. Robust standard errors are clustered at the user level. Coefficients are reported with 95% Confidence Intervals below. ^∗∗∗^p < 0.01, ^∗∗^p < 0.05, ^∗^p < 0.10.*

**Table 4** tests whether different types of side effects are associated with differences in symptom relief. The results are robust across specifications; reporting positive or context-specific side effects is associated with greater symptom relief, while reporting negative side effects is associated with less symptom relief. For example, based on Column (4), a person with a starting symptom level of 5 who reports 100% of negative side effects would experience a 0.5 point increase in symptom severity on a 1–10 scale, whereas a similar user who does not report any negative side effects would experience 2.2 points of symptom relief, highlighting the importance of adjusting for starting symptom severity level and side effect profiles when evaluating the overall effectiveness of cannabis as a treatment modality.

**Table 4:** Association of positive, negative, and context-specific side effects with symptom relief.

Outcome = symptom relief				
	
	(1)	(2)	(3)	(4)
		
	Any {0,1}		Percent of possible in category	
Positive	-1.100^∗∗∗^	-1.344^∗∗∗^	-2.345^∗∗∗^	-2.899^∗∗∗^
	(-1.360 to -0.841)	(-1.578 to -1.111)	(-3.046 to -1.643)	(-3.653 to -2.145)
Negative	0.174^∗∗^	0.336^∗∗∗^	2.311^∗∗∗^	2.772^∗∗∗^
	(0.015 to 0.334)	(0.192 to 0.480)	(1.461 to 3.161)	(2.045 to 3.498)
Context-specific	-0.339^∗∗∗^	-0.239^∗∗∗^	-0.781^∗∗^	-0.417
	(-0.540 to -0.138)	(-0.413 to -0.065)	(-1.495 to -0.068)	(-0.931 to 0.096)
Starting symptom level		-0.660^∗∗∗^		-0.666^∗∗∗^
		(-0.710 to -0.610)		(-0.724 to -0.608)
Constant	-2.307^∗∗∗^	1.894^∗∗∗^	-3.098^∗∗∗^	1.100^∗∗∗^
	(-2.625 to -1.989)	(1.441 to 2.348)	(-3.372 to -2.824)	(0.818 to 1.382)
Observations	10,535	10,535	10,535	10,535
*R*^2^	0.015	0.349	0.036	0.376


*The first two columns measure use the existence of each category of side effect as independent variables, while the second two columns use the percent of possible in each category of side effects. The second and fourth columns include the starting symptom level. In all four regressions, the outcome is the change in symptom severity. Robust standard errors are clustered at the user level. Coefficients are reported with 95% Confidence Intervals below. ^∗∗∗^p < 0.01, ^∗∗^p < 0.05, ^∗^p < 0.10.*

## Discussion

This is the largest observational study to measure immediate changes in patient-reported symptom severity ratings and experienced side effects in real-time from using cannabis under naturalistic conditions. Building on previous research showing that cannabis may be an effective substitute for opioids (Hurd, 2016; Vigil et al., 2017) and other classes of prescription medications (e.g., sedatives; Piper et al., 2017; Stith et al., 2017), we provide evidence that cannabis is used to treat many different types of symptoms for which conventional pharmaceutical medications are typically prescribed, and that the magnitude of reported symptom relief and side effect profiles from using cannabis varies for people with different symptoms. The Releaf App^TM^ users consumed cannabis to treat a wide range of health symptoms, the most frequent relating to pain, anxiety, or depression. Clinically and statistically significant reductions in patient-reported symptom severity levels existed in every single symptom category, suggesting that cannabis may be an effective substitute for several classes of medications with potentially dangerous and uncomfortable side effects and risky polypharmaceutical interactions, including opioids, benzodiazepines, and antidepressants (Weich et al., 2014; Centers for Disease Control and Prevention, 2016; Fontanella et al., 2016; Rudd et al., 2016; Sharma et al., 2016). Higher pre-dosing symptom levels were generally associated with greater post-dosing symptom relief and users treating an anxiety-related symptom or depression showed stronger symptom relief than users treating a pain symptom, even though depression is not a condition approved for medical cannabis use in most states.

Similar to clinical reviews showing that cannabis is associated with numerous, yet generally non-serious side effects (Wang et al., 2008; Whiting et al., 2016), positive and context-specific side effects were more commonly reported than negative side effects by the Releaf App^TM^ users, with the most frequent reported side effects being positive (relaxed, peaceful, comfy) and the least frequent side effects being negative (paranoid, confused, headache). Positive side effect reporting was associated with the greatest reported symptom relief, followed by context-specific side effects, while negative side effects were associated with lower reported symptom relief. In general, patients treating depression were more likely to indicate a negative side effect than patients treating anxiety- or pain-related symptoms, though even users who reported only negative side effects reported significant decreases in moderate to severe symptom intensity levels after using cannabis.

One of the most striking patterns in the current results was the breadth of symptoms that appeared to improve following cannabis consumption. This pattern of responses could have been a function of characteristics of the software user interface (e.g., symptom intensity scale range), manner in which responders interacted with their mobile device (e.g., visual attention to common symptom severity levels), or with the systemic nature by which phytocannabinoids may affect the human mind and body. According to the endocannabinoid deficiency theory, many mental and physical health disturbances result from the dysregulation of the body’s innate endocannabinoid system (ECS; Smith and Wagner, 2014; Di Marzo et al., 2015; Karhson et al., 2016; Russo, 2018), often described as a master network of chemical signals that promote somatic and psychological homeostasis, or psychobiological state-efficiency (Bermudez-Silva et al., 2010; Silvestri and Di Marzo, 2013; Acharya et al., 2017). The ECS consists of natural ligands (e.g., anandamide and 2-AG) and receptors (CB1 and CB2) that appear to play a major role in efficient regulation of a wide range of systems that include sleep, feeding (e.g., gut permeability and adipogenesis), libido and fertility, pain perception, motivation, happiness, anxiety, learning and memory, social functioning, autoimmune responses, cellular redox, and cancer pathophysiology (Valvassori et al., 2009; Muccioli et al., 2010; Abdel-Salam et al., 2012; Cani, 2012; Burstein, 2015; Du Plessis et al., 2015; McPartland et al., 2015; Karhson et al., 2016; Pava et al., 2016; Tegeder, 2016; Turcotte et al., 2016; Androvicova et al., 2017; Sierra et al., 2018). In other words, unlike conventional pharmaceutical approaches, which largely target specific neurotransmitter sites (e.g., monoamine neurotransmitter hypothesis; Delgado, 2000; Ng et al., 2015), cannabis may act to improve a broad spectrum of symptoms by regulating homeostatic functioning, perhaps best described as a system-modulating rather than symptom-modulating form of therapy.

Notwithstanding the strengths of the naturalistic research design and the potential implications of the study’s findings, the study was limited primarily by the lack of a control group, e.g., non-cannabis users with the same symptom using a mobile device to indicate their immediate symptom intensity levels. There is also the potential confound of user-selection bias and exclusion of users that failed to complete sessions or even use the Releaf App^TM^ due to a lack of symptom relief or negative side effects. (It is possible that selection bias could have worked in the opposite way, excluding patients that are already satisfied with their cannabis choices and therefore choose not to use the software app). This study chose to focus on the existence of symptom relief and side effects rather than offer clinical guidance as to which cannabis products offer preferential symptom relief and side effects profiles. As such we did not include product characteristics, e.g., routes of administration, quantity and method of ingestion, and cannabinoid content, all of which are likely crucial for understanding how cannabis affects symptom relief and side effect manifestation. We only show that, on average, most cannabis users experience symptom relief. Future research will benefit by incorporating these contextual factors into measurements of patient decisions and by dissecting how fundamental characteristics of the cannabis products themselves affect immediate and longer term changes in symptom relief and potential adverse consequences.

Patients with certain health conditions such as neurological disorders (e.g., multiple sclerosis, seizures, epilepsy, headache) may face differential risks for experiencing adverse effects or exacerbating their symptoms, for instance, depending on the amount of delta-9-tetrahydrocannabinol they consume, and caution should be used for patients considering using highly potent cannabis products (Solimini et al., 2017). Complicating matters are the allogamous (variable) and unstable nature of the *Cannabis* plant and the inherent inconsistencies in the chemical contents across plant batches and derived formulations, which are affected by genetic characteristics, but also environmental, cultivation, and storage conditions (Thomas and Pollard, 2016; Pacifici et al., 2017, 2018). These factors present challenges for both medical cannabis consumers and researchers as patients never have continuous access to cannabis products with precisely consistent chemotypes. Cannabis-based products (e.g., dried flower vs. oils) can differ in their dose reliability, and researchers have offered guidelines for dosing titration and experimental usage (Kahan et al., 2014; Pichini et al., 2018). However, until federal laws currently restricting pharmacodynamics research in the United States are reformed (Stith and Vigil, 2016) investigators still have tremendous opportunities to develop and incorporate innovative assessment tools, like the Releaf App^TM^, into observational research designs for measuring how patients experience self-directed cannabis treatment in their normal everyday lives outside of clinical settings.

## Author Contributions

JV and SS conceived the study. FB, KK, and BH independently designed and developed the Releaf App^TM^ and server infrastructure as part of their effort to help create an education tool for medical cannabis patients. SS conducted the analyses. JV and SS drafted the manuscript. All authors contributed substantially to its intellectual content and revision.

## Conflict of Interest Statement

The authors FB, KK, and BH were employed by company MoreBetter Ltd. The remaining authors declare that the research was conducted in the absence of any commercial or financial relationships that could be construed as a potential conflict of interest.
