# Supplementary material for: Patient-Reported Symptom Relief Following Medical Cannabis Consumption
Source: Front Pharmacol. 2018 Aug 28;9:916. doi: 10.3389/fphar.2018.00916 (PMC6121171; doi:10.3389/fphar.2018.00916)
Supplement: Supplementary file 1 [file Table_1.DOCX]

SUPPLEMENTAL APPENDIX

Table S1: Descriptive Statistics by Symptom Category

| **Symptom** | **N Users** | **N Sessions** | **Starting Symptom Level** | **Ending Symptom Level** | **Symptom Relief** | **P-Value** |
| --- | --- | --- | --- | --- | --- | --- |
| Agitation / Irritability | 312 | 608 | 6.2 | 1.7 | -4.5 | <0.001 |
| Anxiety | 1027 | 2,344 | 6.3 | 1.9 | -4.4 | <0.001 |
| Convulsions | 19 | 21 | 7.1 | 2.4 | -4.6 | <0.001 |
| Depression | 589 | 1,483 | 6.6 | 2.2 | -4.3 | <0.001 |
| Dizziness | 32 | 50 | 6.9 | 2.6 | -4.3 | <0.001 |
| Excessive Appetite | 41 | 47 | 7.0 | 1.9 | -5.1 | <0.001 |
| Fatigue | 361 | 766 | 6.1 | 2.2 | -3.9 | <0.001 |
| Impulse | 88 | 132 | 5.1 | 1.6 | -3.5 | <0.001 |
| Inflammation | 137 | 281 | 5.7 | 2.7 | -3.0 | <0.001 |
| Insomnia | 399 | 1,012 | 6.8 | 1.9 | -4.9 | <0.001 |
| Loss of Appetite | 123 | 209 | 6.8 | 1.7 | -5.1 | <0.001 |
| Mood Swings | 98 | 129 | 6.0 | 1.8 | -4.2 | <0.001 |
| Muscle Spasms | 27 | 228 | 6.2 | 2.5 | -3.7 | <0.001 |
| Nausea | 169 | 276 | 6.4 | 1.6 | -4.7 | <0.001 |
| Other | 395 | 781 | 5.3 | 1.6 | -3.7 | <0.001 |
| Pain - Abdominal | 86 | 139 | 6.6 | 4.3 | -4.3 | <0.001 |
| Pain - Back | 525 | 1,211 | 6.3 | 2.6 | -3.7 | <0.001 |
| Pain - Cramping | 103 | 172 | 6.6 | 2.2 | -4.4 | <0.001 |
| Pain - Gastrointestinal | 109 | 266 | 6.1 | 2.1 | -4.0 | <0.001 |
| Pain - Headache | 73 | 204 | 5.7 | 2.5 | -3.2 | <0.001 |
| Pain - Joint | 241 | 593 | 6.4 | 2.7 | -3.7 | <0.001 |
| Pain - Migraine | 207 | 381 | 6.4 | 2.4 | -4.0 | <0.001 |
| Pain - Muscle | 258 | 583 | 6.1 | 2.7 | -3.4 | <0.001 |
| Pain - Nerve | 180 | 427 | 6.8 | 3.1 | -3.2 | <0.001 |
| Pain - Other | 185 | 508 | 6.2 | 3.1 | -3.2 | <0.001 |
| Stress | 622 | 1,357 | 5.8 | 1.7 | -4.1 | <0.001 |
| Tremors | 16 | 17 | 6.1 | 1.6 | -4.5 | <0.001 |
| Wellness | 66 | 264 | 4.8 | 1.0 | -3.9 | <0.001 |

Notes: Includes all data for which both starting and ending symptoms were recorded and the starting symptom level was greater than 0.
