# Supplementary material for: Patient-Reported Symptom Relief Following Medical Cannabis Consumption
Source: Front Pharmacol. 2018 Aug 28;9:916. doi: 10.3389/fphar.2018.00916 (PMC6121171; doi:10.3389/fphar.2018.00916)
Supplement: Supplementary file 2 [file Table_2.DOCX]

Table S2: Descriptive Statistics – Side Effects

| **Side Effect** | **% Sessions Reporting** | **Symptom Relief** | **p-value** | **Category** |
| --- | --- | --- | --- | --- |
| Active | 6% | -4.1 | <0.001 | Positive |
| Anxious | 11% | -3.3 | <0.001 | Negative |
| Chill | 29% | -3.7 | <0.001 | Positive |
| Clear | 23% | -4.7 | <0.001 | Positive |
| Comfy | 38% | -4.1 | <0.001 | Positive |
| Confused | 4% | -3.4 | <0.001 | Negative |
| Couchlocked | 18% | -4 | <0.001 | Context-Specific |
| Creative | 14% | -4.3 | <0.001 | Positive |
| Distracted | 12% | -3.4 | <0.001 | Context-Specific |
| Dizzy | 11% | -3.6 | <0.001 | Negative |
| Dreamy | 33% | -4 | <0.001 | Positive |
| Dry Mouth | 23% | -3.7 | <0.001 | Negative |
| Energetic | 14% | -4 | <0.001 | Positive |
| Focused | 22% | -3.8 | <0.001 | Positive |
| Foggy | 22% | -3.7 | <0.001 | Negative |
| Forgetful | 13% | -3.6 | <0.001 | Negative |
| Frisky | 10% | -3.9 | <0.001 | Positive |
| Grateful | 14% | -4.3 | <0.001 | Positive |
| Great | 21% | -4.2 | <0.001 | Positive |
| Happy | 20% | -4 | <0.001 | Positive |
| Headache | 4% | -3.2 | <0.001 | Negative |
| High | 33% | -3.9 | <0.001 | Context-Specific |
| Hungry | 21% | -3.7 | <0.001 | Context-Specific |
| Irritable | 5% | -3.1 | <0.001 | Negative |
| Light | 25% | -4.1 | <0.001 | Positive |
| Optimistic | 16% | -4.2 | <0.001 | Positive |
| Paranoid | 3% | -3.1 | <0.001 | Negative |
| Peaceful | 54% | -4 | <0.001 | Positive |
| Productive | 10% | -3.9 | <0.001 | Positive |
| Red Eyes | 10% | -3.9 | <0.001 | Negative |
| Reflective | 25% | -3.9 | <0.001 | Positive |
| Relaxed | 64% | -3.9 | <0.001 | Positive |
| Restless | 11% | -3.4 | <0.001 | Negative |
| Scattered | 10% | -3.2 | <0.001 | Negative |
| Silly | 8% | -4.3 | <0.001 | Context-Specific |
| Sleepy | 27% | -3.8 | <0.001 | Context-Specific |
| Talkative | 12% | -4 | <0.001 | Context-Specific |
| Thirsty | 26% | -3.9 | <0.001 | Context-Specific |
| Tingly | 19% | -4 | <0.001 | Context-Specific |
| Tuned | 22% | -4.1 | <0.001 | Positive |
| Unmotivated | 11% | -3.3 | <0.001 | Negative |
| Visuals | 4% | -3.7 | <0.001 | Context-Specific |

Notes: Includes all data for which both starting and ending symptoms were recorded and the starting symptom level was greater than 0.
