# Supplementary figures and images for: Patient-Reported Symptom Relief Following Medical Cannabis Consumption
Source: Front Pharmacol. 2018 Aug 28;9:916. doi: 10.3389/fphar.2018.00916 (PMC6121171; doi:10.3389/fphar.2018.00916)

Figure S1. Frequency of Levels of Symptom Relief

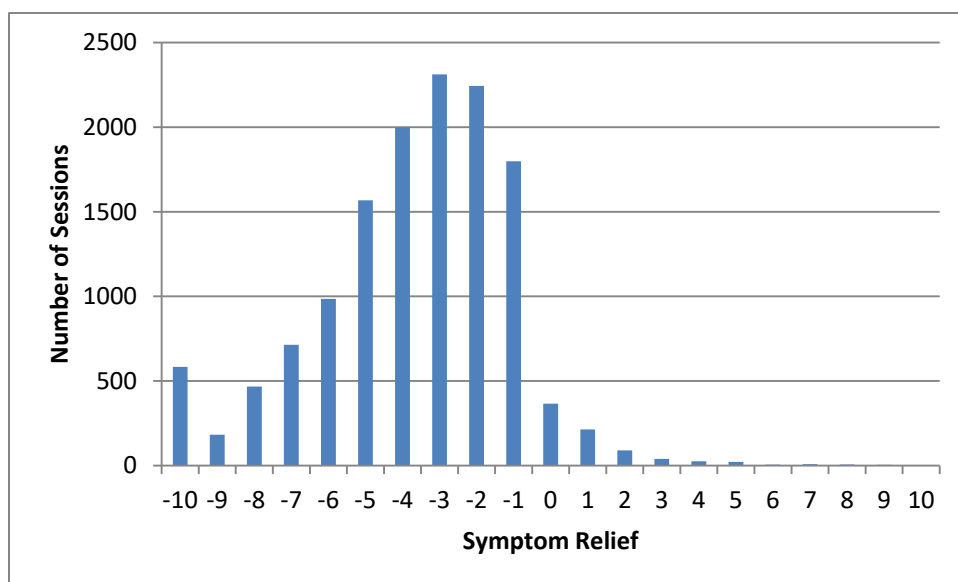

Supplement: Supplementary file 3 [file Image_1.pdf]

Figure S2. Symptom Relief by Starting Symptom Level and Symptom Category

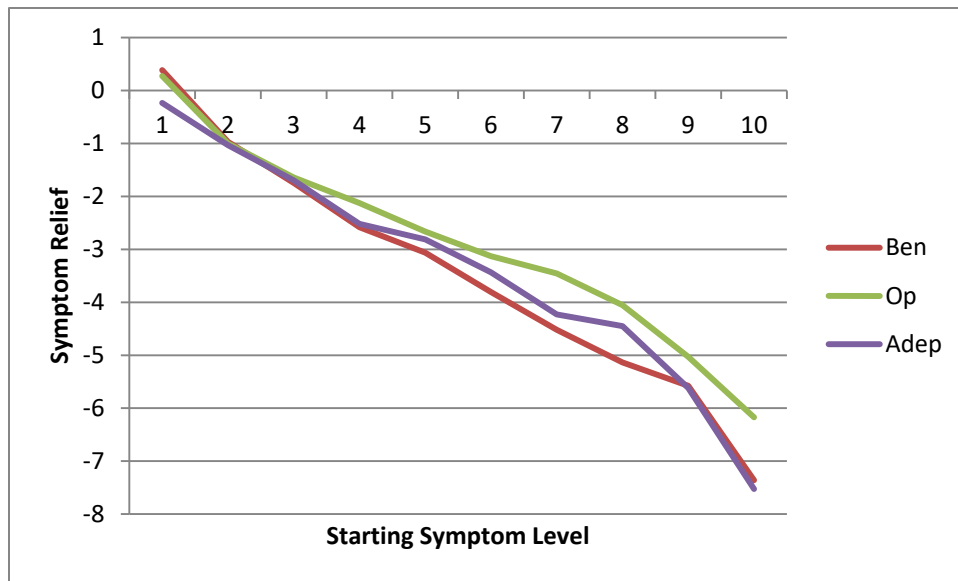

Supplement: Supplementary file 4 [file Image_2.pdf]
